# Operationalizing Next-Generation Sequencing in a Community-Based Academic Cancer Center: Workflow, Integration, and Impact

**DOI:** 10.3390/cancers18030534

**Published:** 2026-02-06

**Authors:** Gayathri Moorthy, Annette Sereika, Bruce Brockstein, Megan Parilla, Mir B. Alikhan, Michael Bouma, Janardan Khandekar, Dyson Wake, Peter J. Hulick, Henry M. Dunnenberger, Linda Sabatini, Mathew Yang, Kathy A. Mangold, Erin Proctor, Nicholas Evans, Nicholas Miller, Donald L. Helseth, Darryck Maurer, Justin Brueck, Karen Kaul

**Affiliations:** 1 Neaman Center for Personalized Medicine, Evanston, IL 60201, USA; annette.sereika@endeavorhealth.org (A.S.); janardan.khandekar@endeavorhealth.org (J.K.); dyson.wake@endeavorhealth.org (D.W.); peter.hulick@endeavorhealth.org (P.J.H.); mark.dunnenberger@endeavorhealth.org (H.M.D.); mathew.yang@endeavorhealth.org (M.Y.); erin.proctor@wfusm.edu (E.P.); nicholas.evans@endeavorhealth.org (N.E.); nicholas.miller@endeavorhealth.org (N.M.); larry.helseth@endeavorhealth.org (D.L.H.J.); darryck.maurer@endeavorhealth.org (D.M.); justin.brueck@endeavorhealth.org (J.B.); 2 Division of Hematology/Oncology, Endeavor Health, Evanston, IL 60201, USA; bruce.brockstein@endeavorhealth.org; 3 Department of Pathology and Laboratory Medicine, Endeavor Health, Evanston, IL 60201, USA; megan.parilla@endeavorhealth.org (M.P.); mir.alikhan@endeavorhealth.org (M.B.A.); michael.bouma@endeavorhealth.org (M.B.); linda.sabatini@endeavorhealth.org (L.S.); kathy.mangold@endeavorhealth.org (K.A.M.); karen.kaul@endeavorhealth.org (K.K.)

**Keywords:** real-world study, next-generation sequencing, precision oncology, pharmacogenomics, bioinformatics, molecular tumor board, genomic profiling, implementation science, NGS

## Abstract

**Background/Objectives**: Prompt integration of molecular and clinical data into electronic medical records, with a sustainable workflow that supports clinicians in rendering genomics-guided care, is critical. We sought to expand the implementation of in-house NGS at our community-based academic cancer center to operationalize the utilization of molecular diagnostic studies to optimize cancer care for all patients, including those outside this study, through broader adoption and diffusion. **Methods**: In this prospective IRB-approved study, the Kellogg Cancer Genomic Initiative (KCGI), patients with advanced cancers underwent in-house NGS, including tumor mutational burden (TMB) and pharmacogenomics. In-house bioinformatics (Flype) was used for structured reporting and served as a molecular knowledgebase. A multidisciplinary molecular tumor board (MTB) was created to provide precision therapy recommendations. **Results**: In-house NGS, completed within 11 business days on average, was performed in 90% (251) of the 279 patients in the KCGI with advanced cancers. RNA and TMB analyses were successful in 89.2% and 86.5% of patients, respectively. A total of 54.2% of patients were identified as candidates for use of on- or off-label FDA-approved therapies, and 99.6% of patients who underwent pharmacogenomics testing had at least one gene alteration associated with medication dose adjustment/avoidance. An MTB was established to discuss these and other molecularly challenging cases continues to function as a consultative service that provides actionable recommendations. **Conclusions**: In this real-world trial, the utilization of in-house NGS with an adaptable bioinformatics pipeline and the establishment of an MTB enabled the refinement of institutional processes and created an environment that enhanced clinician interest in genomics and improved genomics-guided care for patients with advanced cancers.

## Simple Summary

This prospective study, the Kellogg Cancer Genomic Initiative, sought to expand the implementation of in-house next-generation sequencing (NGS) and pharmacogenomics within a community-based academic cancer center. Among 279 enrolled patients with advanced cancers, in-house NGS was performed in 90%, with an average turnaround of 11 business days. Over half of patients who had NGS were identified as candidates for NGS-guided therapies, and the majority had pharmacogenomic variants relevant to treatment management. Additionally, by establishing an in-house bioinformatics pipeline and a multidisciplinary molecular tumor board, we enhanced institutional workflows, clinician engagement, and precision oncology care delivery. The KCGI culminated in the successful adoption and integration of personalized data within our community health system.

## 1. Introduction

Molecular studies such as pharmacogenomics and somatic next-generation sequencing (NGS) of tumors are fundamental for diagnosis, prognosis and treatment decision-making in cancer. The field is evolving rapidly, and multiple commercial and academic laboratories provide these services. NorthShore University HealthSystem (part of the newly formed Endeavor Health), a large multi-institutional system in northern suburban Chicago, implemented the in-house NGS of tumor tissues and pharmacogenomics over a decade ago and strives to improve these services for our patients with cancer. Our in-house molecular testing includes a smaller panel of 50 genes for limited samples and an expanded panel reporting on 158 genes with broader analysis to evaluate the tumor mutation burden (TMB). The laboratory also performs a 10-gene RNA fusion panel [1]. This testing approach has enabled the NorthShore molecular laboratory to keep up with the increasing numbers of targeted therapeutics, some with tumor-agnostic indications.

To better deliver and utilize advanced molecular testing at NorthShore/Endeavor, we undertook a prospective observational trial, known as the Kellogg Cancer Genomic Initiative (KCGI). We sought to expand implementation of in-house NGS at our community-based academic cancer center to operationalize the utilization of molecular diagnostic studies and optimize cancer care for all patients, including those outside this study, through broader adoption and diffusion. Though the KCGI, we refined our processes and capacities to provide care for patients with cancer in this study and, by diffusion, in the routine care of patients. The KCGI was financially supported by philanthropy via the Mark R. Neaman Center for Personalized Medicine’s Transformation through Innovation Fund and led by the Medical Oncology and Pathology Departments of NorthShore/Endeavor with input from a multidisciplinary team from oncology, molecular pathology, medical genetics, bioinformatics, pharmacogenomics, and nursing.

**Figure 1:**
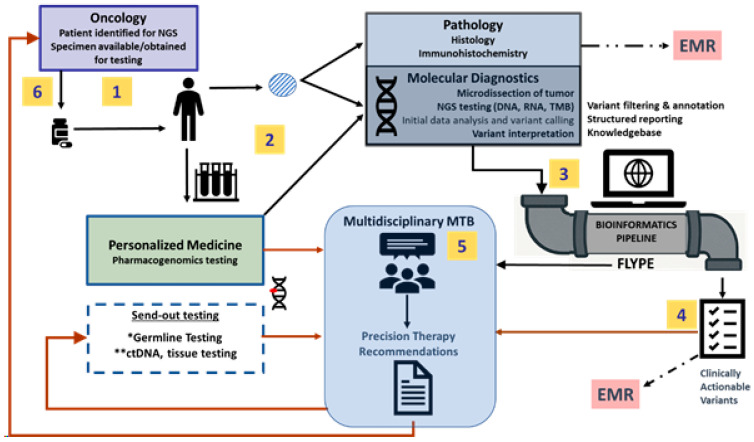
Model of genomics-guided care at NorthShore/Endeavor that leverages in-house molecular diagnostics, bioinformatics, personalized medicine, and a multidisciplinary molecular tumor board (MTB) in a sustainable workflow that supports precision oncology. Patients are identified (1) by the treating oncologist as candidates for molecular diagnostic studies such as NGS and pharmacogenomics testing (2). With bioinformatics support (Flype) (3), variants are filtered, interpreted, annotated, and reported in a structured format (4) that is integrated within the electronic medical record (EMR). A multidisciplinary team of providers discuss these somatic molecular results along with results of in-house pharmacogenomics testing and send-out testing, including germline testing (*) and circulating tumor DNA (** ctDNA), to offer precision therapy recommendations within a comprehensive report (5). The report is sent to the treating oncologist who can act on therapy (6) or other testing recommendations.

In this paper, we describe the molecular capabilities and bioinformatics structure that allowed for the rapid analysis of data. We describe the development of a sustainable workflow for clinicians and a molecular tumor board (MTB) (Figure 1). The framework may serve as a model for the successful integration of genomic data into community-based cancer care. The clinical outcomes of NGS testing through the KCGI will be described in a separate manuscript.

## 2. Materials and Methods

### 2.1. Enrollment

The KCGI is a single-center, prospective, institutional review board (IRB)-approved observational study that enrolled adult patients with advanced or metastatic non-hematological cancers treated at the NorthShore Kellogg Cancer Centers. These patients were identified by their treating oncologist because NGS might alter treatment beyond standard of care. Patients were consented/enrolled in protocol EH20-392 “Prospective analysis of the utility of routine incorporation of genomic panels into the care of patients with metastatic cancer”, approved by the NorthShore IRB. Patients had formalin-fixed paraffin-embedded tissue or cytology smears available for molecular analysis; new samples were obtained only if needed for pathologic assessment. Patients who previously had tumor NGS as part of routine clinical care could be enrolled for additional sequencing if newer biomarkers on the NorthShore panel were not included on the test previously performed or if there was interest in identifying molecular evolution on a new specimen.

### 2.2. NGS Analysis

**Figure 2:**
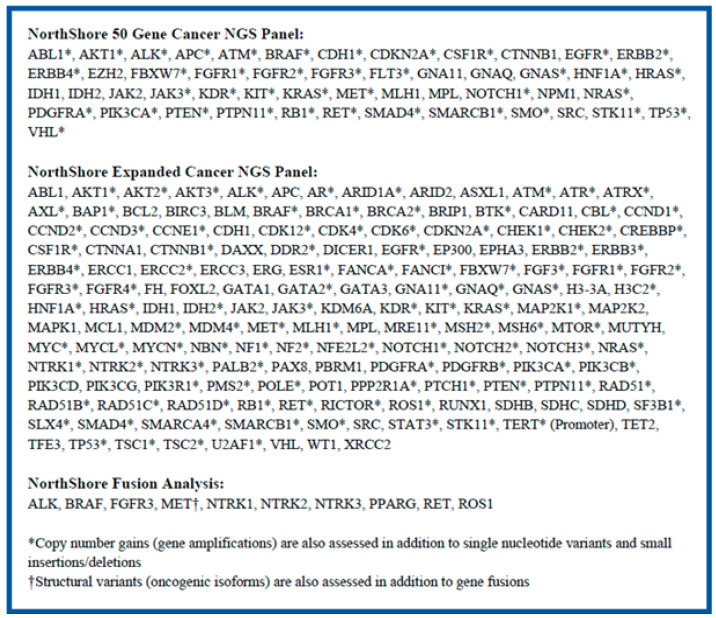
Genes included in the in-house NGS testing with the NorthShore Expanded Cancer NGS Panel (158 genes and TMB) and the Northshore 50-gene Cancer NGS Panel (50 genes, without TMB), and fusion analysis. Select genes were also analyzed for (*) copy number gain/amplification analysis and (†) structural variant analysis.

DNA analysis: The CLIA-validated NorthShore Expanded NGS panel was performed for solid tumor samples from enrolled patients. This panel assessed 1.7 Mb DNA and reported out mutations in 158 cancer-associated oncogenes and tumor suppressor genes (Figure 2). This extensive analysis also supported the evaluation of TMB status and copy number gains (gene amplifications). If the specimen was insufficient to perform the Expanded panel, the 50-gene NGS panel was performed (Figure 2). Sequencing was performed on an IonTorrent S5 platform. Initial data analysis, alignment to the reference genome, and variant calling were conducted with the on-board Torrent Suite software (v5.8). The aligned files were transferred to our in-house bioinformatics pipeline, Flype [starting at v6.3 (03/17/2020) now upgraded to v10.6], for filtering, annotation, and variant reporting [2]. Variant interpretation was based on multiple national databases (e.g., Ensembl’s VEP, CIViC, cBioPortal, OncoKB, COSMIC, NCI TP53 database, ClinVar, BRCA exchange, My Cancer Genome, JAX CKB, PMKB, MD Anderson), NCCN guidelines, CAP/ASCO/AMP guidelines, and a literature review [3,4,5,6,7,8,9,10,11,12,13,14,15,16,17,18]. Additional information regarding the specifics of the two DNA panels is available in Supplemental Document S1.

RNA analysis: Translocation analysis by RNA sequencing was performed on all samples that passed RNA quality metrics to identify translocations or oncogenic isoforms in *ALK*, *ROS*, *RET*, *MET*, *NTRK1*, *NTRK2*, *NTRK3*, *FGFR3*, *BRAF*, and *PPARG* by NGS (Figure 2). Fusion testing involved RNA isolation from the tumor, reverse transcription into cDNA, and target selection with a custom ArcherDx panel. Sequencing was again performed on the IonTorrent platform. Alignment and variant calling were performed with locally run Archer DX software (Version 6.2.7). Data were then transferred to Flype for variant classification, annotation, and reporting. Additional information regarding the specifics of this RNA panel is available in Supplemental Document S1.

Pharmacogenomics: Whole blood was collected for pharmacogenomics assessment using a CLIA-validated multi-gene pharmacogenomic panel. Testing methodology included primer extension and mass spectrometry, quantitative real-time PCR, restriction fragment length polymorphism analysis, and fragment analysis by capillary electrophoresis [19]. Genes associated with impact on chemotherapy (*DPYD*, *UGT1A1*, *TPMT*), pain management (*CYP2C9*, *CYP2D6*, *OPRM1*), mental health (*CYP2B6*, *CYP2D6*, *CYP2C19*, *SLC6A4*), cardiovascular system (Factor II, Factor V), and other health conditions were included. Clinical guidance was provided through a summary report, integrated clinical decision support, and direct inclusion of relevant results within the MTB note.

### 2.3. Reporting/Bioinformatics

NorthShore/Endeavor’s bioinformatics team developed an in-house bioinformatics pipeline, known as Flype, to review, interpret, and sign-out results for a variety of genetic tests [2]. The NGS report signed out by one of our molecular pathologists was incorporated directly from Flype into the EMR as a PDF linked to the pathology report for that specimen. The pharmacogenomics testing was also reported via Flype after review by pharmacists. Flype converts raw variant calls into “star alleles”. The data are then transferred to the EMR as both an easy-to-understand PDF and as discrete data for use with in-house-built clinical decision support tools triggered by electronic prescriptions [20]. Flype, NorthShore’s web-based genomics platform interfaced with Epic [2], was also utilized to prepare individualized notes summarizing the patient’s clinical history; their somatic, germline, and pharmacogenomics results with attention to actionable findings; as well as clinical recommendations from the multidisciplinary MTB. Supplementary figures and images can be incorporated to enhance the recommendation provided by the report. Generic information about the genomic results may also be saved to a knowledge base to be leveraged in similar future MTB cases.

Flype was built using open-source tools including the popular Python (v3.9)-based web application framework, Django. In partnering with NorthShore/Endeavor’s Health Information Technology group, who maintain dedicated Linux servers, the bioinformatics team was provided a dedicated team of database administrators for support and data backup. This allowed the small team to have an increased impact. The size of the bioinformatics team has fluctuated over the course of ten years from two to five bioinformaticians (2 to 4.5 full-time-equivalents). Although the original Flype architecture, using Django (v4.2), was developed with separate software modules, which had the potential for portability, recent enhancements and customizations—including the development of a laboratory information system (LIS)—limits its ability to be used in other settings. The additional software changes will be detailed in a forthcoming publication.

### 2.4. Molecular Tumor Board

We developed a nearly real-time MTB using our genomic knowledgebase within Flype. The MTB team comprised oncology-certified advanced practice nurses, clinical specialists in pharmacogenomics, molecular pathologists, bioinformaticians, medical geneticists, and oncologists. The team reviewed data for each patient referred to the MTB or enrolled in the KCGI prior to the presentation and discussion at the monthly MTB. The MTB consultative service was available for all patients, and sessions were broadly attended and included learners. Pertinent clinical/oncology history, results of all available somatic and germline testing performed in-house and externally, pharmacogenomics results, and MTB recommendations were summarized in MTB notes that were shared electronically with the treating oncologists. Oncologists had the option of incorporating these notes into the EMR, in addition to storage within Flype.

### 2.5. Operations

During the KCGI, we sought to optimize all aspects of the program, including ordering of tests, reporting, consultation and establishment of an MTB, and use of the output data. In addition, prior authorization, billing, and reimbursement were recorded, but the complicated analyses of these financials are still ongoing.

## 3. Results

### 3.1. Benefit Investigation

Of the 279 participants enrolled, benefit investigation was not performed for the 106 patients (38%) with Medicare. Of the 173 participants with commercial insurance who had benefit investigations, 36 were approved (20.8%), 53 were denied (30.6%), and 1 patient had no benefits for genetic testing. No prior authorizations were required for 83 patients (48.0%). Regardless of the benefit investigation result, the claim was sent to the insurance provider. The cost of testing, if not covered by insurance, was borne by this study. Actual reimbursement analyses are ongoing.

### 3.2. NGS Ordering

After enrollment in this study, NGS was ordered using the “molecular pathology add-on order” by a study member directly within the EMR (Epic). This triggered an adequacy review of archival material by an anatomic pathologist who estimated tumor purity and assisted in selecting the NGS panel based on the amount of tumor in the specimen. A statement regarding the adequacy of the specimen, type of NGS testing underway, and expected turn-around time was communicated via specialized reports into Epic within the “Personalized Medicine” dashboard built to inform the clinical team the status of testing and facilitate finding results. The average turn-around time (TAT) from the time the specimen was received at the molecular pathology laboratory to the time NGS was reported was 11 business days. The calculated median TAT from order to result was 10 business days. The estimated median TAT from laboratory receipt of specimen to report was 8 business days. The ability of the laboratory to deliver results rapidly and to utilize cytology and small biopsy samples were the primary advantages of using in-house testing capabilities.

### 3.3. KCGI Enrollment and NGS Metrics

**Figure 3:**
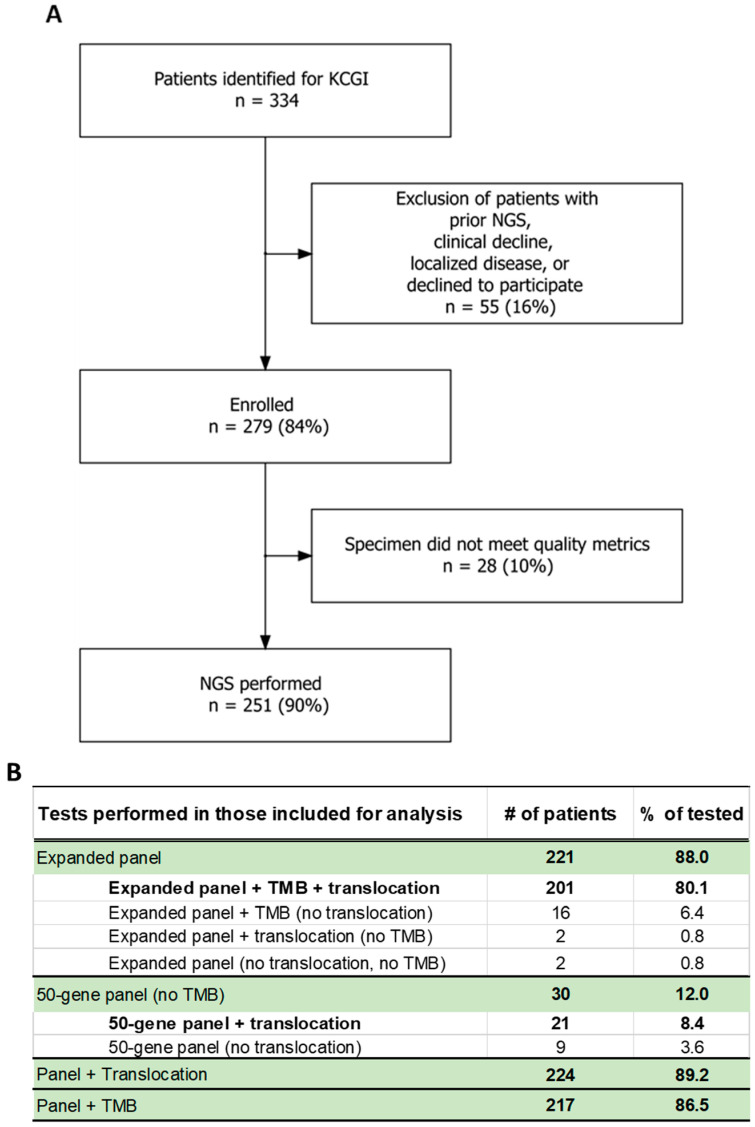
(**A**) KCGI enrollment and exclusion statistics and (**B**) testing statistics.

The KCGI enrollment algorithm and testing metrics are presented in Figure 3. Of the 279 patients who participated in this study, 251 patients (75.1% of identified and 90.0% of enrolled) had NGS performed (Figure 3A). A total of 221 patients (88.0%) had the 158-gene Expanded panel, and 30 patients had the limited (50-gene) panel (Figure 3B). NGS was unsuccessful in 10% of the 279 patients and was limited to the 50-gene panel in 12% of those who underwent testing, due to low tumor purity (<20% tumor nuclei), insufficient sample (scant or exhausted tissue blocks), or nucleic acid damage due to specimen processing (e.g., acid-based decalcification of bone specimens). Pancreatic cancer represented 11 of 15 patients (73.3%) who had the 50-gene panel, as pancreatic resection specimens typically have low tumor purity, and the biopsies are limited (small and/or few passes by fine-needle aspiration). The expanded panel included TMB analysis, which was successful in all but four patients (1.6%). The TMB was blinded/removed from the report when the specimens had poor-quality sequencing metrics (i.e., low total coding coverage) [21]. Translocation/fusion testing was successful in 89.2% of the 251 patients tested but failed in 27 due to RNA quality, typically due to sample age (specimens older than 2 years may have degraded RNA). The sequencing failure rate in this study is comparable to the 8–11% reported in previous studies that utilized Foundation Medicine, in the NCI-MATCH trial that utilized the Oncomine platform, and other MATCH-certified laboratories [22,23,24,25].

### 3.4. Summary of Actionable Alterations and Targeted Therapies Identified by NGS

**Figure 4:**
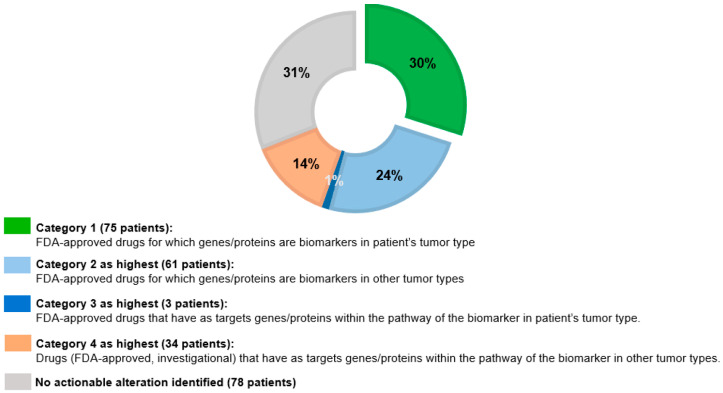
Proportion of patients (of the 251 who had NGS testing) with no actionable alteration and with alterations in each category (1–4). Of note, the highest level of actionability for each patient was determined. Actionable alterations were prioritized for the level of actionability into one of four categories, with category 1 alterations being the most actionable and category 4 alterations being the least actionable. The majority of patients in the KCGI had actionable alteration(s) identified through in-house NGS.

The details regarding the study outcomes including actionable alterations and targeted therapies are described in a separate outcomes report and summarized in Figure 4. A total of 204 (81.3%) of the 251 patients tested had at least one clinically relevant tier I/II alteration identified as designated by ASCO/AMP/CAP [18]. As of June 2025, 173 patients (68.9%) had alterations that could be targeted with drugs that were either approved for use, could be used off-label, or were investigational (Figure 4). A total of 75 patients (29.9%) had at least one alteration qualifying them for an FDA-approved drug for their tumor type; the majority of these patients had breast cancer, uterine cancer, and thyroid cancer (of note, lung and colorectal cancer, which have high rates of actionable alterations, are poorly represented in this study as NGS is performed routinely for those cases at our institution). Of those without FDA-approved therapy options in their tumor type, 61 patients (24.3%) had alterations that predict benefit from FDA-approved drugs used off-label. Eight patients (3.2%) had fusions identified. Treatment options, targeting genes in a variety of cellular pathways, were identified in patients based on the results of NGS testing, including tumor-agnostic therapies such as pembrolizumab, inhibitors of NTRK, RET, and BRAF/MEK, and fam-trastuzumab deruxtecan in 40 patients (15.9%) with a variety of tumor types. 

### 3.5. Pharmacogenomics Testing Results

Of the 279 patients enrolled, 251 patients (75.1% of identified and 90.0% of enrolled) had pharmacogenomics testing performed, and all but one (99.6%) had at least one gene alteration associated with a recommendation to alter treatment for at least one medication. The median number of genes with variants predicted to impact medication dosing, toxicity, or efficacy was five. Impact on pain management including ibuprofen, meloxicam, hydrocodone, and tramadol was the most common, with 169 patients (67%) having alterations that could interact with such therapy. A potential impact on chemotherapy medications such as tamoxifen, fluorouracil, irinotecan or azathioprine was discovered in 118 patients (47%). The number of patients with impact on medications for mental health and cardiovascular concerns was similar: 123 patients (49%) had a gene variant that impacted antidepressant or antianxiety medications, and 121 (48%) had a result that indicated an interaction with anticoagulation, cholesterol management, or antiarrhythmics.

### 3.6. Molecular Tumor Board Presentations

All patients enrolled in the KCGI with successful NGS were discussed at the monthly MTB sessions. Physicians also referred patients outside the trial to the MTB for discussion of in-house or external genomic/genetics testing. Prior to each MTB, a team comprising a clinical pharmacist, advanced practice nurses, an oncologist, and a molecular pathologist met to discuss cases and prepared individualized reports. Reports were prepared using Flype, which served as a knowledge base for up-to-date pathologist-verified variant information and as a platform for MTB presentations of cases.

**Figure 5:**
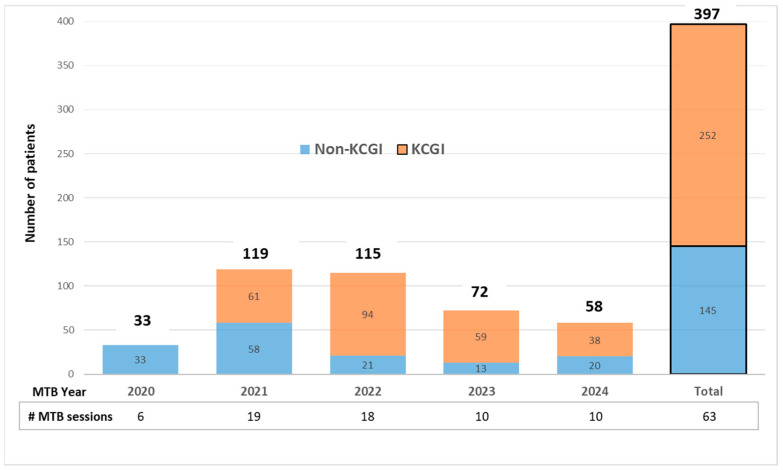
Numbers of patients presented at the MTB from 2020 through 2024 stratified by whether they were participants in the KCGI or referred to the MTB by oncologists outside of the trial (non-KCGI).

From July 2020 through 2024, we presented 397 patients in a total of 63 MTB sessions, with more than a third being referred to the MTB outside of the trial (“non-KCGI”, Figure 5). The MTB included a multidisciplinary team of 8–18 attendees in person or virtually via a HIPAA-compliant platform. Recommendations made by the team were not fundamentally different from the consensus reached by the multidisciplinary MTB. The MTB recommendations were communicated via Flype reports to oncologists. Oncologists utilized these recommendations to support the selection of precision therapies for patients with actionable alterations. The KCGI and/or clinical team was responsible for facilitating appeals for precision therapies. There is evidence presented by others on the value of MTBs in determining appropriate molecular tests, interpreting results, and recommending precision therapies, leading to improved patient outcomes [26,27]. As the survival data from the KCGI patients were immature, we identified 33 patients with advanced cancers who received NGS-identified therapies, 19 of whom experienced responses for more than 3 months on these therapies.

### 3.7. Identification of Patients Who May Be Candidates for Germline Genetic Testing

The involvement of genetics providers in the MTB review process was instrumental in identifying patients needing referral for germline testing based on personal and family history and/or the presence of certain somatic variants at an appropriate allele frequency, as shown by others [28]. Both the ESMO Precision Medicine Working Group and ASCO have identified 40 genes that, when seen in particular circumstances in somatic tissue testing, should prompt further germline testing [29,30]. Of the 251 patients who underwent NGS, 31 met ESMO/ASCO criteria for the consideration of germline testing based on the somatic variant identified. More than half of these patients either had no prior germline testing (14 patients) or limited prior germline testing (3 patients). Population-based germline testing through Endeavor Health’s Genetic Wellness Assessment has been ongoing at our institution for several years, and the institution has a robust genetic cancer risk assessment program within the personalized medicine department that is well-poised to serve our oncology patients who would benefit from risk assessment and germline testing [31].

## 4. Discussion

Barriers to providing comprehensive personalized cancer care have been enumerated in other papers, as have methods for the validation of similar in-house NGS and pharmacogenomics assays [32,33,34,35]. This study is a unique resource, however, in its comprehensive discussion of solutions, specifically detailing the components found in the successful adoption and integration of personalized data in a community-based academic cancer center. In this narrative summary, our institution chronicles the resources and tools—mobilized through a research study—that resulted in sustained clinician uptake and identified potentially actionable biomarkers in over 50% of patients. A comparable effort could be undertaken in other similar-sized hospital systems in the United States using this research model as a framework. The applicability to other regions of the world is, however, limited.

Recognizing the importance of molecular diagnostics in precision oncology, NorthShore/Endeavor has prioritized the development of in-house NGS and bioinformatics support tools with integration into EMRs following the framework for a genomics learning healthcare system [36,37]. Through the KCGI, a framework was established for patients with advanced cancers to receive NGS and pharmacogenomics testing with TAT of 2–3 weeks and expert consultation in real time through a multidisciplinary MTB attended by our molecular pathologists. This sustainable workflow for genomics-guided care can be replicated in other similar systems; 85% of cancer care is provided in community-based institutions like ours [38].

The in-house NGS panels utilized were comparable to those provided by commercial vendors (published TAT for one major vendor was 8 days from receipt in laboratory to report) [39]. Both the 50-gene and 158-gene expanded NGS panels have been optimized for low-input specimens with amplicon-based enrichment that requires much less DNA compared with the capture-based enrichment methods used in most commercial laboratories. Ultimately, the success rate of our in-house panel (Figure 3B) was comparable that of commercial laboratories while allowing for testing of scant cytology samples [22,25]. A total of 30% of patients who underwent 50-gene panel testing had actionable alterations (seven of whom had category 1 alterations as the highest recommendation and two of whom had category 2 as the highest). These patients would have not benefited from the value of NGS if low-DNA-input specimens were rejected. Commercial vendors offer more genes and broader translocation panels, but many of these genes are not currently actionable. The limitations of our assays include the inability to detect copy number losses/most gene deletions and, at the time of the study, FGFR1 and FGFR2 fusions.

The value of in-house NGS and bioinformatics is highlighted in the outcomes of the KCGI, where over 50% of the patients were identified as candidates for FDA-approved precision oncology therapies either on- or off-label. The integration of in-house NGS results within the EMRs allows easy access to information for clinical decision-making. The bioinformatics tool Flype further facilitates variant interpretation, updating, and reporting and enables comparison of NGS results obtained at different times. Over the course of the KCGI from 2021 through 2024, there were at least 10 new drugs approved that require assessment of a DNA/RNA-based biomarker [8,40]. The Flype pipeline is powered to enable easy identification of patients harboring select variants that may become actionable over time as more precision therapies are approved or enter into clinical trials. The conclusions from this study are dynamic, as guideline-driven standard-of-care recommendations tend to change over time. These processes, along with the multidisciplinary MTB service, promote effective collaborations between molecular pathology, oncology, medical genetics, pharmacogenomics, and the research team as well as the diffusion of genomics knowledge to MTB participants. Of note, while the numbers of patients presented at the MTB decreased (partly due to fewer KCGI participants), we had an increase in non-KCGI participants at the MTB, which continues monthly at our institution.

An additional aim of the KCGI was to develop a sustainable workflow for clinicians from the ordering of in-house molecular testing to the utilization of this information for the identification of appropriate therapies. Over the course of this study, the structure of our molecular pathology and HIT processes as well as the fundamental bioinformatics structured reporting provided by Flype were modified to incorporate clinician preferences and promote clinician uptake. The creation of a dashboard that provided live NGS status updates and linked to in-house and external genomic testing results, pharmacogenomics results, and germline testing results aided in improving the workflow for clinicians. The MTB was valuable not only for attendees but also provided scientific support for selecting targeted therapies. With success of the NGS and MTB within NorthShore/Endeavor, we plan to expand these services to providers and patients across our broader health system.

## 5. Conclusions

In conclusion, through the KCGI, we were able to expand the implementation and value of in-house NGS and pharmacogenomics to patients with advanced cancers treated within our community-based academic cancer center. We developed a sustainable workflow for clinicians from the ordering of in-house molecular testing to the utilization of results for the identification of appropriate therapies, with support from a consultative MTB. Our molecular pathology, HIT, and bioinformatics pipeline (Flype) processes incorporated clinician preferences to promote uptake. Ultimately, the tools and processes developed within this research study allowed the expansion of personalized medicine across our broader health system.

## Data Availability

The data are contained within this article. Additional data are available in a separate manuscript submitted for publication and available upon reasonable request.

## Supplementary Materials

The following supporting information can be downloaded at: https://www.mdpi.com/article/10.3390/cancers18030534/s1. References [41,42] are cited in the Supplementary Materials.

## Abbreviations

The following abbreviations are used in this manuscript:

KCGI	Kellogg Cancer Genomic Initiative
NGS	Next-generation sequencing
TMB	Tumor mutational burden
MTB	Molecular tumor board
IRB	Institutional review board
EMR	Electronic medical record
TAT	Turn-around time
