# Supplementary material for: Operationalizing Next-Generation Sequencing in a Community-Based Academic Cancer Center: Workflow, Integration, and Impact
Source: Cancers (Basel). 2026 Feb 6;18(3):534. doi: 10.3390/cancers18030534 (PMC12897014; doi:10.3390/cancers18030534)
Supplement: Supplementary file 1 [file cancers-18-00534-s001.zip › cancers-4138543-supplementary.pdf]

Supplemental Document 1

Clinical Validation of the NorthShore Expanded Cancer NGS Panel

Summary: The NorthShore Expanded Cancer NGS Panel is a 158-gene targeted next-generation sequencing assay that enables the detection of SNVs, indels, and copy number gains in relevant oncogenes and tumor suppressor genes. This assay also provides the ability to report tumor mutation burden by including 1.155Mb of coding coverage (and now also microsatellite instability status). This assay utilizes DNA extracted from FFPE tissue and Papanicolaou / DiffQuik stained cytology smears after macro-dissection.

Sequencing platform: ION Torrent (Ion-Semiconductor Sequencing)

Panel: The ThermoFisher Oncomine Comprehensive Assay v3 is used in conjunction with the Oncomine Tumor Mutation Load Assay to evaluate a total of 158 cancer-associated oncogenes and tumor suppressor genes. Variant calling is a combination of platform-specific Ion Torrent Software and in-house validated pipelines; annotation and reporting is assisted by an in-house bioinformatics platform [1,2]

Validation Methods: Accuracy was established primarily using patient samples (FFPE and cytology smears) that were sequenced on clinically validated NGS pipelines (validated platforms included: Foundation Medicine and the NorthShore Ion AmpliSeq Cancer HotSpot Panel). CAP proficiency testing samples and reference standards were also used to assist in the validation of MSI and TMB. Sixty-two unique samples were run for sensitivity and specificity of variant calling; these were comprised entirely of patient samples. For run-to-run and instrument-to-instrument reproducibility two patient samples were used on both machines. For within-run reproducibility one patient sample was run 4 times. For technologist-to-technologist reproducibility, three patient samples were run by two different medical laboratory scientist (MLS) team members. This analysis yielded a 100% concordance of all 2900 variants with a MAF average SD of 1.27%. MSI was validated using an additional 96 samples: 93 patient samples (49 compared to Foundation Medicine NGS MSI, 44 to clinically validated Promega MSI PCR), two proficiency CAP samples, and 1 horizon diagnostics sample. TMB was analyzed with 65 unique samples (63 patient samples all compared to foundation medicine; 2 FFPE reference standards from SeraCare). Copy number methods and validation is detailed in a separate publication [1].

Tumor Diagnosis/Type	Number of Samples Tested	
Lung	17	
Colorectal	20	
Breast	1	
Melanoma	6	
Ovarian/ GYN	3	
Brain	2	
Prostate	0	
Sarcoma	0	
Thyroid	2	
Bladder / Upper Tract Urothelial	2	
Other / Unknown	9	
*Contrived	0	
*Reference Material	0	
	62	Total ***clinical*** samples in validation
	62	Total samples in validation

Validation Results:

Single Nucleotide Variant: Sensitivity: 99.7% / Specificity: 100%
Insertion/Deletion Variants: Specificity: 96% / Specificity: 100%
Copy Number Variants (single gene; amplifications): Sensitivity of 92.3%/ Specificity of 99.9%

Sample requirements for SNV/CNV/Indel: 35% tumor purity*
Sample requirements for TMB: 35% Tumor purity
Sample requirements for MSI: 40% tumor purity

Note: our copy number threshold is 2.8-fold to call a gene an unequivocal amplification.

> * OCA assay requires 20% tumor purity; the TML assay requires 35% tumor purity. Samples with tumor purity between 20-35% are thus typically reflexed to the 50-gene panel except in unique circumstances.

The correlation plot below for Foundation Medicine-TMB and NorthShore-TMB (Figure below) shows a regression R2 value of 0.88 with a Slope of 0.69. These values are in concordance with the Friends of Cancer Research harmonization project where correlation studies between panel-TMB and WES-TMB show a regression R2 range of 0.77 – 0.96 with slopes ranging 0.60 – 1.26 (1).

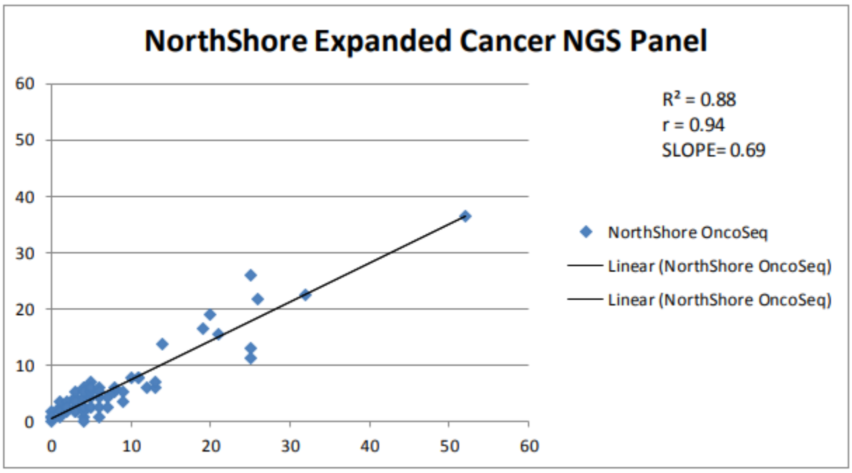

Variant Annotation, Filtering and Interpretation:

ɨVEP release 96 is used for variant annotation. Version (GRCh37)

This includes constituent databases / references:
Ensembl database version: 109
Genome assembly GRCH37.p13
GENCODE 19
RefSeq 2015-01
Regulatory build 1.0
PolyPhen: 2.2.2
SIFT: 5.2.2
dbSNP 151
COSMIC 86

HGMD-PUBLIC 2017.4
ClinVar 2018-10
1000 Genomes Phase 3
NHLBI-ESP V2-SSA137
gnomADg r2.1, exomes only

Manual data review is done in Integrative Genomics Viewer (IGV).

Tiering of variants / assessment of oncogenicity is based on ClinGen/CGC and VICC guidelines in conjunction with AMP/ASCO guidelines [3,4]. Primary databases utilized in automatic filtering include: gnomAD, VEP and ClinVar. For population frequency filtering gnomAD is used with a 1% VAF Max frequency filter. To ensure high frequency pathogenic variants are caught a "pull back" mechanism utilized the ClinVar database: a ClinVar status of pathogenic, likely pathogenic or conflicting interpretations of pathogenicity allows the variant to supersede the 1% "maxfreq" population filter. Additionally, functional effect /predictive data as determined by VEP (specifically deeply intronic >10bp from exon and synonymous variants) are filtered with the same ClinVar database pull back rules as above to capture likely pathogenic / pathogenic splicing variants).

Manual tiering was based on several databases / tools. Functional data was captured by OncoKB or medical /scientific literature searches. Cancer hotspots utilized COSMIC and cancerhotspots.org. Computational databases included SIFT, alphamissense, and Polyphen2. CBioportal was also used for on-tumor frequencies – specifically the databases for TCGA and GENIE. NCCN was used as a reference in the interpretive write-up. Please note: this is not an exhaustive list of all possible databases used in uncommon / unique circumstances.

<u>Clinical Validation of the NorthShore Fusion Analysis Next-Generation Sequencing Assay</u>

Summary: The NorthShore Fusion Analysis next-generation sequencing assay targets 10 oncogenes of known clinical significance. This panel uses RNA extracted from FFPE tissue and Papanicolaou / DiffQuik stained cytology smears after macro-dissection.

Sequencing platform: ION Torrent (Ion-Semiconductor Sequencing)

Panel: Fusion testing is performed with a custom Archer FusionPlex Panel targeting 10 genes. Fusion variant calling is done by Archer Analysis software with reporting assisted by our in-house bioinformatics platform [2].

Validation Methods: 46 clinical / patient samples, 8 contrived specimens (7 CAP samples and one positive control sample from Horizon Diagnostics) and 3 reference standards (Horizon Diagnostics reference Standard v1, Horizon Diagnostics reference Standard, and SeraSeq NTRK fusion reference standard) were used in the validation of fusions. The clinical samples were all compared to clinically validated NGS or FISH testing methods from (Foundation Medicine, NeoGenomics, Thyroseq, GlioSeq, University of Chicago, Mayo Clinic MN, and UPMC/University of Pittsburgh) or clinically validated single-gene FISH/ fusion testing methods at NorthShore. For run-to-run and instrument-to-instrument reproducibility both patient samples and reference standards were run by two different MLS team members in three independent runs. This analysis yielded a 100% concordance.

Tumor Diagnosis/Type	Number of Samples Tested	
Lung	13	
Colorectal	1	
Breast	0	
Melanoma	0	
Ovarian/ GYN	0	
Brain	12	
Prostate	0	
Sarcoma	1	
Thyroid	12	
Bladder	0	
Other/Unknown	7	
*Contrived	8	
*Reference Material	3	
	46	Total ***clinical*** samples in validation
	57	Total samples in validation

Validation Results:
Fusions: Specificity: 100% / Specificity: 100%

Sample requirements: 20% tumor purity.

Variant Annotation and Interpretation:
Fusion variant calling is done by Archer Analysis software with reporting assisted by our in-house bioinformatics platform [2]. Quality filtering is as follows: minimal number of break point spanning reads to call a valid fusion must be 5. The minimal unique start sites to call a valid fusion must be 3. The minimal percentage of fusion reads per GSP2 must be 10. Each sample must pass Fusion QC which is set to a cutoff of 10 mean unique RNA start sites per control GSP2. Manual tiering of clinical significance was based on several databases / tools: Functional data was captured by OncoKB or medical / scientific literature searches. CBioportal was also used for on-tumor frequencies – specifically the databases for TCGA and GENIE. NCCN was used as a reference in the interpretive write-up.

Note: Archer fusion testing was initially designed for lung and thyroid targets; however, as it covers the majority of medically targetable fusions, it has been an invaluable resource for advanced cancers. In 2025 the genes *THADA*, *FGFR1* and *FGFR2* were validated and added to the panel (after the completion of the KCGI). The addition of *FGFR1* and F*GFR2* has made this panel a comprehensive fusion panel for the identification of fusion-associated biomarker therapies.

**Gene**	**Primary Cancer Associations (DEX)**	**Associated FDA -approved Therapies (any tumor type)**
ALK	Solid tumors, all	crizotinib, ceritinib, alectinib, brigatinib, lorlatinib, ensartinib
RET	Lung, Thyroid	selpercatinib, pralsetinib
ROS1	Lung	crizotinib, entrectinib, repotrectinib and taletrectinib
MET	Lung	capmatinib and tepotinib
FGFR3	Bladder	erdafitnib
NTRK1	Solid tumors, all	larotrectinib, entrectinib and repotrectinib
NTRK2	Solid tumors, all	larotrectinib, entrectinib and repotrectinib
NTRK3	Solid tumors, all	larotrectinib, entrectinib and repotrectinib
BRAF	CNS*	tovorafenib
PPARG	Thyroid	none

* Not found in DEX (https://www.dexzcodes.com/) validation requirements

Clinical Validation of the NorthShore Cancer Hotspot Panel

Summary: The NorthShore Cancer Hotspot panel is a 50-gene targeted next-generation sequencing assay that enables the detection of SNVs, indels, and copy number gains in relevant oncogenes and tumor suppressor genes. This assay cannot report microsatellite instability status or tumor mutation burden. This assay utilizes DNA extracted from FFPE tissue and Papanicolaou / DiffQuik stained cytology smears after macro-dissection.

Sequencing platform: ION Torrent (Ion-Semiconductor Sequencing)

Panel: The assay utilizes the IonTorrent S5/GeneStudio Ion AmpliSeq Cancer HotSpot Panel v2. Variant calling is a combination of platform-specific Ion Torrent Software and in-house validated pipelines; annotation and reporting is assisted by an in-house bioinformatics platform [1,2].

Validation Methods: Accuracy was established primarily using patient samples (FFPE and cytology smears) that were tested on clinically validated platforms - comparator testing included clinically validated PCR or sanger sequencing (NorthShore, ACL laboratories, Oregon Health and Science University) and clinically validated NGS testing (Foundation Medicine, University of Chicago, Caris, Response Genetics, University of Pittsburg Medical center). In addition to patient samples, one Horizon Diagnostics reference standard was also used. Forty-six unique samples were run for sensitivity and specificity; these were comprised entirely of patient samples. Run-to-run reproducibility was performed on 3 samples. Combined there were an observed 47 total variants, all with 100% concordance with a MAF average SD of 1.3%. Within-run reproducibility was performed on 2 samples that included 42 variants 39 SNV and 1 deletion, 1 insertion, and 1 indel, all with 100% concordance. Copy number methods and validation is detailed in a separate publication [1].

Tumor Diagnosis/Type	Number of Samples Tested	
Lung	23	
Colorectal	6	
Breast	0	
Melanoma / skin	3	
Ovarian / GYN	0	
Brain	1	
Prostate	0	
Sarcoma	1	
Thyroid	7	
Bladder	0	
Other / Unknown	5	
*Contrived	0	
*Reference Material	1	
	46	Total ***clinical*** samples in validation
	47	Total samples in validation

Validation Results:
Single Nucleotide Variant: Sensitivity: 98.0% / Specificity: 100%
Insertion/Deletion Variants: Specificity: 91% / Specificity: 100%
Copy Number Variants (Single gene; amplifications): Sensitivity of 100% / Specificity of 100%

Specimen requirements for SNV/CNV/Indel: 20% tumor purity

Note: our copy number threshold is 2.8-fold to call a gene an unequivocal amplification.

Variant Annotation, Filtering and Interpretation:

ƗVEP release 96 is used for variant annotation. Version (GRCh37)

This includes constituent databases / references:
Ensembl database version 109
Genome assembly GRCH37.p13
GENCODE 19
RefSeq 2015-01
Regulatory build 1.0
PolyPhen 2.2.2
SIFT 5.2.2
dbSNP 151
COSMIC 86
HGMD-PUBLIC 2017.4
ClinVar 2018-10
1000 Genomes Phase 3
NHLBI-ESP V2-SSA137
gnomADg r2.1, exomes only

Manual data review is done in Integrative Genomics Viewer (IGV).

Tiering of variants / assessment of oncogenicity is based on ClinGen/CGC and VICC guidelines in conjunction with AMP/ASCO guidelines [3,4]. Primary databases utilized in automatic filtering include: gnomAD, VEP and ClinVar. For population frequency filtering gnomAD is used with a 1% VAF Max frequency filter. To ensure high frequency pathogenic variants are caught a “pull back” mechanism utilized the ClinVar database: a ClinVar status of pathogenic, likely pathogenic or conflicting interpretations of pathogenicity allows the variant to supersede the 1% “maxfreq” population filter. Additionally, functional effect /predictive data as determined by VEP (specifically deeply intronic >10bp from exon and synonymous variants) are filtered with the same ClinVar database pull back rules as above to capture likely pathogenic / pathogenic splicing variants).

Manual tiering was based on several databases / tools. Functional data was captured by OncoKB or medical /scientific literature searches. Cancer hotspots utilized COSMIC and cancerhotspots.org. Computational databases included SIFT, alphamissense, and Polyphen2. CBioportal was also used for on-tumor frequencies – specifically the databases for TCGA and GENIE. NCCN was used as a reference in the interpretive write-up. Please note: this is not an exhaustive list of all possible databases used in uncommon / unique circumstances.

ƗNote: VEP96 was used when we started KCGI then switched to VEP109 on 5/9/23 and VEP111 on 4/23/24.

References

1. Miller, N.; Bouma, M.; Sabatini, L.; Gulukota, K. SILO: A Computational Method for Detecting Copy Number Gain in Clinical Specimens Analyzed on a Next-Generation Sequencing Platform. *J Mol Diagn* **2021**, *23*, 1241–1248, doi:10.1016/j.jmoldx.2021.07.016.
2. Helseth, D.L., Jr.; Gulukota, K.; Miller, N.; Yang, M.; Werth, T.; Sabatini, L.M.; Bouma, M.; Dunnenberger, H.M.; Wake, D.T.; Hulick, P.J.; et al. Flype: Software for enabling personalized medicine. *Am J Med Genet C Semin Med Genet* **2021**, *187*, 37–47, doi:10.1002/ajmg.c.31867.
3. Horak, P.; Griffith, M.; Danos, A.M.; Pitel, B.A.; Madhavan, S.; Liu, X.; Chow, C.; Williams, H.; Carmody, L.; Barrow-Laing, L.; et al. Standards for the classification of pathogenicity of somatic variants in cancer (oncogenicity): Joint recommendations of Clinical Genome Resource (ClinGen), Cancer Genomics Consortium (CGC), and Variant Interpretation for Cancer Consortium (VICC). *Genet Med* **2022**, *24*, 1991, doi:10.1016/j.gim.2022.07.001.
4. Li, M.M.; Datto, M.; Duncavage, E.J.; Kulkarni, S.; Lindeman, N.I.; Roy, S.; Tsimberidou, A.M.; Vnencak-Jones, C.L.; Wolff, D.J.; Younes, A.; et al. Standards and Guidelines for the Interpretation and Reporting of Sequence Variants in Cancer: A Joint Consensus Recommendation of the Association for Molecular Pathology, American Society of Clinical Oncology, and College of American Pathologists. *J Mol Diagn* **2017**, *19*, 4–23, doi:10.1016/j.jmoldx.2016.10.002.
